# Supplementary material for: Acceptance and compliance with micronutrient powder (MNP) among children aged 6–23 months in northern Nigeria
Source: PLOS Glob Public Health. 2022 Oct 17;2(10):e0000961. doi: 10.1371/journal.pgph.0000961 (PMC10022258; doi:10.1371/journal.pgph.0000961)
Supplement: S1 Dataset — (ZIP) [file pgph.0000961.s008.zip › S8_Dataset/Spot Checks.zip!/Spot Checks/05072015_A_SO_PM_2_SC a.pdf]

# SPOT CHECK RECORDING FORM

**State: Adamawa LGA: ____Song____ Community: _Wurokoncha_**

**Name of caregiver (female): Nana Yakubu**

**Name or caregiver (male): ………………………**

**Sex of index child: _female___ Age of index child (in months): _ 8 months_____________**

## Spot Check Information

1. **Date of MNP distribution to household** (circle option below based on LGA name above)**:**
   - **Kebbi:** BirninKebbi&Jega LGA: **June 16**
   - **Kebbi:** Danko-wasagu: **June 17**
   - **Adamawa:**Song, Ganye: **(June 18)**
   - **Adamawa:**Guyuk **June 19**
2. **Date of Spot Check Observation: ____5/7/2015_____**
3. **Number of sachets remaining**(Ask the caregiver to show you and count the specific number of sachets remaining)**:**

   42

4. **Number of sachets that should remainusing one MNP sachet per day** (Analyst only)**:**

   44

5. **Number of sachets remaining / number of sachets should be remaining**(Analyst only)**:**

   **_____42____ / ___44______ = 95.5____ %**

## Probes

**Question 1.** You have **XX** MNP sachets remaining. Explain how easy or difficult it was to use the MNP product on a daily basis for your child 6 – 23 months? (Circle one answer)

**Very Difficult** **Difficult** **(Easy)** **(Very Easy)**

**Question 2.**Describe any specific challenges that you faced that made the MNP difficult to use on a daily basis?

None

**Question 3.** Describe specific aspects of the MNP product that you or your child enjoyed that made it easier to use on a daily basis?

- I notice that the baby sleeps more often
- I feel the baby is receiving additional care to grow properly
- I also feel the MNP will prevent some minor sickness from attacking the child

**Question 4.** What questions do you have about the MNP based on your experience using it?

- If these ones am using (MNP) is exhausted where can I get more
- If I mix the MNP inside the baby food and she did not finish the food at that time, will I still feed her the food mixed with MNP later or what should I do?

**Question 5.** What specific recommendations do you have that we can do as a program to make the MNP easier to use on a daily basis?

If there can be jingles on radio to remind most mothers not to forget to give their baby's the MNP on a daily basis
